# Draft Genome Sequence of a Putative Raw Milk-Associated Probiotic Bacterium, Pediococcus acidilactici ISO17

**DOI:** 10.1128/mra.00208-23

**Published:** 2023-05-11

**Authors:** Goitsemang Makete, Tshifhiwa Paris Mamphogoro

**Affiliations:** a Gastro-Intestinal Microbiology and Biotechnology Unit, Agricultural Research Council-Animal Production, Irene, Pretoria, South Africa; University of Maryland School of Medicine

## Abstract

Pediococcus acidilactici is a beneficial lactic acid bacterium frequently studied for its probiotic potential. Here, we present a 2.32-Mb draft genome sequence of strain ISO17, with a G+C content of 42%. The genome was predicted to harbor 4 rRNA genes, 47 tRNA genes, and 2,297 protein-coding sequences.

## ANNOUNCEMENT

Lactic acid bacteria (LAB) are commonly utilized as probiotics across host species (1). Pediococcus acidilactici is a lactic acid bacterium frequently used as a starter culture in the dairy industry for the production of healthy functional foods, such as cheese and yogurt; the strain is also applicable in other food industries for its probiotic potential (2, 3). To confirm its probiotic characteristics, LAB strain should stimulate immunomodulation against pathogens in the gastrointestinal tract (GIT), survive in the bile and acidic conditions, and adhere to the epithelial cells (4, 5).

Strain ISO17 was isolated from raw milk samples of South African Saanen goats breed collected at the Small-Stock Division of the Agricultural Research Council-Animal Production (Irene, South Africa; 25°53′59.6″S, 28°12′51.6″E). Bacterial cells were obtained by diluting 1 mL of each milk sample into 9 mL of sterile 0.85% saline solution. The organism was cultured on de Man, Rogosa and Sharpe (MRS) solid agar plates supplemented with 0.05 g/L cysteine-HCl (MRS-cysHCl) and incubated anaerobically using an Anaerobic Gas Pack (Oxoid) for 24 to 48 h at 37°C (5). The genome of ISO17 was extracted using a Quick-DNA miniprep kit (Zymo Research, Irvine, CA, USA) following the manufacturer’s instructions, and the genomic DNA library was prepared using the NEBNext Ultra II FS DNA library prep kit (New England Biolabs, Ipswich, MA). This library was sequenced on the Illumina NextSeq platform at Inqaba Biotechnical Industries (Pty) Ltd. (Pretoria, South Africa), producing a total of 1,204,990 paired-end, 2 × 150-bp reads. The raw Illumina paired-end reads were filtered to remove low-quality reads and adapter regions using Trimmomatic v0.36 (6), with a minimum quality score of 15 and a minimum sequence length of 70. The quality of the reads was evaluated using FastQC v0.11.5 (7). The trimmed reads were then used for *de novo* assembly using SPAdes v3.13.0 (8) via the KBase platform (9). Contigs smaller than 500 bp, typically representing sequencing artefacts, were removed, and the filtered assembly was used for the subsequent analysis. Genome assembly statistics were generated using QUAST (10). The completeness and contamination of the genome were assessed using CheckM v1.0.18 (11). Default parameters were used for all software tools employed in the analysis.

ISO17 was identified as Pediococcus acidilactici using Kaiju v1.7.3 (12), and the results were visualized using Krona v2.7.1 (13). Genome assembly of this strain revealed a genome size of 2,321,968, an *N*_50_ length of 31,032 bp, an average G+C content of 42%, and 156× genome coverage. Genome annotation was carried out using the Rapid Annotations Subsystems Technology (RAST) (14) and the NCBI Prokaryotic Genome Annotation Pipeline (PGAP) v6.3 (15), resulting in 290 contigs with 2,297 coding sequences, 55 RNAs, 4 rRNA genes, 47 tRNA genes, and 32 pseudogenes. Rapid *in silico* analysis of the secondary metabolite biosynthesis gene clusters using antiSMASH v6.0.0 (16) and RAST revealed the presence of genes involved in bile salt tolerance, acid tolerance, adhesion to epithelial cells, antioxidant production, and immunomodulation against pathogens (Table 1), all of which are linked to probiotic activities (4, 17).

**TABLE 1:** Genes related to probiotic characteristics in the genome of Pediococcus acidilactici ISO17

Function	Locus tag	NCBI protein accession no.	Annotation
Acid tolerance	PWH42_00475	MDD9322494.1	F0F1 ATP synthase subunit epsilon
	PWH42_00480	MDD9322495.1	F0F1 ATP synthase subunit beta
	PWH42_00485	MDD9322496.1	F0F1 ATP synthase subunit gamma
	PWH42_00490	MDD9322497.1	F0F1 ATP synthase subunit alpha
	PWH42_00495	MDD9322498.1	ATP synthase F1 subunit delta
	PWH42_00500	MDD9322499.1	F0F1 ATP synthase subunit B
	PWH42_00505	MDD9322500.1	F0F1 ATP synthase subunit C
	PWH42_00510	MDD9322501.1	F0F1 ATP synthase subunit A
	PWH42_00865	MDD9322570.1	Cochaperone GroES
	PWH42_00870	MDD9322571.1	Chaperonin GroEL
	PWH42_01625	MDD9322703.1	Na+/H+ antiporter NhaC family protein
	PWH42_03175	MDD9323008.1	Na+/H+ antiporter NhaC
	PWH42_05505	MDD9323464.1	Sodium/proton antiporter
	PWH42_09585	MDD9324247.1	Cation/proton antiporter
Antioxidant	PWH42_00180	MDD9322436.1	Thioredoxin
	PWH42_00350	MDD9322470.1	Thiol peroxidase
	PWH42_00985	MDD9322593.1	Thioredoxin-disulfide reductase
	PWH42_01605	MDD9322699.1	Catalase
	PWH42_02920	MDD9322957.1	Peptide-methionine (S)-*S*-oxide reductase MsrA
	PWH42_02925	MDD9322958.1	Peptide-methionine (R)-*S*-oxide reductase MsrB
	PWH42_03560	MDD9323084.1	Arsenate reductase family protein
	PWH42_04235	MDD9323214.1	Glutaredoxin-like protein NrdH
	PWH42_06620	MDD9323684.1	Thioredoxin family protein
	PWH42_07080	MDD9323765.1	Pyruvate oxidase
Adhesion	PWH42_00630	MDD9322523.1	Glucose-6-phosphate isomerase
	PWH42_01055	MDD9322606.1	Type I glyceraldehyde-3-phosphate dehydrogenase
	PWH42_01065	MDD9322608.1	Triose-phosphate isomerase
	PWH42_01070	MDD9322609.1	Phosphopyruvate hydratase or enolase
	PWH42_01510	MDD9322680.1	Elongation factor Tu
	PWH42_05240	MDD9323412.1	Tyrosine-protein phosphatase
	PWH42_06015	MDD9323565.1	Class A sortase
	PWH42_08100	MDD9323964.1	LPXTG cell wall anchor domain-containing protein
	PWH42_10165	MDD9324362.1	CpsD/CapB family tyrosine-protein kinase
	PWH42_10350	MDD9324395.1	Tyrosine protein phosphatase
Bile salt tolerance	PWH42_02945	MDD9322962.1	manganese-dependent inorganic pyrophosphatase
	PWH42_03120	MDD9322997.1	GNAT family *N*-acetyltransferase
Immunomodulation	PWH42_05000	MDD9323364.1	d-Alanine–poly(phosphoribitol) ligase subunit DltA
	PWH42_05005	MDD9323365.1	d-Alanyl-lipoteichoic acid biosynthesis protein DltB
	PWH42_05010	MDD9323366.1	d-Alanine–poly(phosphoribitol) ligase subunit DltC
	PWH42_05015	MDD9323367.1	d-Alanyl-lipoteichoic acid biosynthesis protein DltD

### Data availability.

This whole-genome shotgun sequencing project has been deposited at DDBJ/ENA/GenBank under the accession number JAQZRB000000000. The version described in this paper is the first version. The SRA accession number is SRR23215526, the BioProject accession number is PRJNA896361, and the BioSample accession number is SAMN32902060.
